# Pharmacologic Comparison of Clinical Neutral Endopeptidase Inhibitors in a Rat Model of Acute Secretory Diarrhea[Fn FN3]

**DOI:** 10.1124/jpet.115.231167

**Published:** 2016-05

**Authors:** David W. Griggs, Michael J. Prinsen, Jonathan Oliva, Mary A. Campbell, Stacy D. Arnett, Deena Tajfirouz, Peter G. Ruminski, Ying Yu, Brian R. Bond, Yuhua Ji, Georg Neckermann, Robert K. M. Choy, Eugenio de Hostos, Marvin J. Meyers

**Affiliations:** Center for World Health and Medicine, Saint Louis University, St. Louis, Missouri (D.W.G, M.J.P, J.O., M.A.C., S.D.A., D.T., P.G.R., M.J.M); Gateway Pharmacology Laboratories, St. Louis, Missouri (Y.Y, B.B); and PATH, San Francisco, California (Y.J., G.N., R.K.M.C, E.dH.)

## Abstract

Racecadotril (acetorphan) is a neutral endopeptidase (NEP) inhibitor with known antidiarrheal activity in animals and humans; however, in humans, it suffers from shortcomings that might be improved with newer drugs in this class that have progressed to the clinic for nonenteric disease indications. To identify potentially superior NEP inhibitors with immediate clinical utility for diarrhea treatment, we compared their efficacy and pharmacologic properties in a rat intestinal hypersecretion model. Racecadotril and seven other clinical-stage inhibitors of NEP were obtained or synthesized. Enzyme potency and specificity were compared using purified peptidases. Compounds were orally administered to rats before administration of castor oil to induce diarrhea. Stool weight was recorded over 4 hours. To assess other pharmacologic properties, select compounds were orally administered to normal or castor oil–treated rats, blood and tissue samples collected at multiple time points, and active compound concentrations determined by mass spectroscopy. NEP enzyme activity was measured in tissue homogenates. Three previously untested clinical NEP inhibitors delayed diarrhea onset and reduced total stool output, with little or no effect on intestinal motility assessed by the charcoal meal test. Each was shown to be a potent, highly specific inhibitor of NEP. Each exhibited greater suppression of NEP activity in intestinal and nonintestinal tissues than did racecadotril and sustained this inhibition longer. These results suggest that newer clinical-stage NEP inhibitors originally developed for other indications may be directly repositioned for treatment of acute secretory diarrhea and offer advantages over racecadotril, such as less frequent dosing and potentially improved efficacy.

## Introduction

Acute secretory diarrhea (ASD) is characterized by rapid onset and life-threatening loss of water and electrolytes. An estimated 1.7 billion episodes of ASD occur each year, resulting in the deaths of an estimated 580,000 children younger than 5 years old, mostly in the developing world (UNICEF, 2013; Fischer Walker et al., 2012). There is also a substantial burden of morbidity and mortality in older children, adolescents, and adults (Lamberti et al., 2014). Survivors frequently contend with multiple, recurrent episodes of ASD associated with additional long-term consequences such as increased susceptibility to infections, malnutrition, and delayed mental development. Oral rehydration therapy is widely accepted as an essential approach to prevent mortality, but administration of fluids alone does not provide quick clinical relief of symptoms; this has led to proposals that compliance and efficacy of oral rehydration therapy may be increased in some settings by cotreatment with an agent that attenuates intestinal hypersecretion. Ideally, such a drug would work rapidly when conveniently delivered not more than once daily and would not delay intestinal transit to avoid concerns about pathogen retention, reactive constipation, or abdominal pain and bloating.

Opioid receptors, especially those of the *μ* and *δ* subtypes, regulate intestinal motility and fluid secretion in an overlapping but pseudoselective manner (Galligan and Akbarali, 2014; Thompson et al., 2014). The body’s endogenous opioid ligands for these receptors are the enkephalins, which attenuate cAMP and alter other second-messenger pathways to decrease secretion. Enkephalins are normally degraded within minutes of release by local peptidases but can be stabilized by pharmacologic inhibition of the enzyme neutral endopeptidase (NEP), also known as enkephalinase or neprilysin (Giros et al., 1987; Khaket et al., 2012). This widely expressed metalloprotease is synthesized in a membrane bound form, and its enzymatic activity can be easily detected in rodent tissues, as shown previously (Giros et al., 1987; Spillantini et al., 1990) and in this report.

Besides enkephalins, many other small secreted peptides are purported substrates of NEP, including atrial natriuretic peptide, endothelin-1, substance P, bradykinin, glucagon-like peptide, angiotensin-1, gastrin, secretin, vasoactive intestinal peptide (VIP), neurotensin, neuropeptide Y, and amyloid *β* (Turvill and Farthing, 1997; Skidgel and Erdos, 2004). Because these peptides are implicated in regulating a wide range of physiologic and pathologic states, NEP has been a target of significant interest to pharmaceutical developers, which have advanced a number of potent NEP inhibitors to human clinical trials.

Only one NEP inhibitor is approved and marketed in select countries for the treatment of ASD: racecadotril (acetorphan). The compound is a lipophilic diesterified prodrug that is rapidly converted by tissue esterases to its active metabolite, thiorphan. Efficacy of orally administered racecadotril was first characterized in a rat model of castor oil–induced diarrhea (Marcais-Collado et al., 1987). Efficacy was reversed by treatment with an opiate receptor antagonist, supporting the drug’s mechanism of action as stabilization of enkephalin signaling. Subsequent success was reported from many small randomized single- or double-blinded placebo-controlled ASD clinical trials in which racecadotril was orally administered to adults and children (Matheson and Noble, 2000; Lehert et al., 2011; Eberlin et al., 2012). Both animal and human data showed treatment does not affect gastrointestinal transit time (Marcais-Collado et al., 1987; Bergmann et al., 1992), and diarrhea patients treated with racecadotril were less likely to develop reactive constipation and other side effects than those treated with the antimotility agent loperamide (Prado, 2002; Wang et al., 2005; Eberlin et al., 2012).

Despite its apparent benefits, racecadotril suffers from a suboptimal pharmacokinetic profile such that the standard therapeutic dosing regimen is 100–300 mg three times daily (Matheson and Noble, 2000). Frequent dosing, along with its intensely bitter taste, arguably modest efficacy, and other factors may all combine to diminish patient compliance and restrain enthusiasm for greater use; however, many other NEP inhibitors have progressed in recent years to clinical studies for pain, hypertension, congestive heart failure, and sexual disorders. These drugs, which can be expected to have varied physicochemical, pharmacokinetic (PK) , and pharmacodynamic (PD) properties, have, to the best of our knowledge, not been tested for antidiarrheal efficacy. Since activity in the rat castor oil–induced diarrhea model was previously shown to be successfully translated to activity for racecadotril in human ASD trials, we performed studies to directly compare efficacy of several clinical-stage NEP inhibitors in this model. The most promising compounds were then further compared for dose-, concentration-, and time-dependent effects on NEP enzyme inhibition and for undesired effect on intestinal motility.

## Materials and Methods

### 

#### Test Compounds.

Racecadotril and thiorphan were purchased from Toronto Research Chemicals (Toronto, ON) and Santa Cruz Biotechnology (Santa Cruz, CA), respectively. Candoxatril and candoxatrilat were provided by Pfizer Inc. (New York, NY). Omapatrilat and gemopatrilat were provided by Bristol-Myers Squibb (New York, NY). Ilepatril and MDL 100240 were provided by Sanofi (Paris, France). The University of Missouri-St. Louis Medicinal Chemistry Group was contracted to synthesize UK-505,749. WuXi AppTec was contracted to synthesize sacubitril, also known as AHU-377, by a procedure modified from that described by Ksander et al. (1995). LBQ657, the active metabolite of sacubitril, was synthesized by hydrolysis of sacubitril with sodium hydroxide (Ksander et al., 1995) and purified by reverse-phase high-performance liquid chromatography. The sodium salts of sacubitril and candoxatril were obtained by treatment of solutions of the test compounds in tetrahydrofuran with one equivalent of 1 N sodium hydroxide (Ksander et al., 1995), evaporation of tetrahydrofuran, and lyophilization to provide the sodium salts as white powders.

#### Purified Peptidase Assays.

Purified recombinant human enzymes were purchased from R&D Systems (Minneapolis, MN). The quenched peptide substrate for aminopeptidase N was Ala-AMC (Bachem 1410, Torrance, CA). The substrate for puromycin-sensitive aminopeptidase was Leu-AMC (Bachem 1240). The substrate for all remaining enzymes was MCA-RPPGFSAFK-Dnp-OH (R&D Systems). Each substrate was diluted to 100 *μ*M in the assay buffer recommended for each enzyme by the supplier. To a black microtiter plate (Corning 3686), 5 *μ*l of compound dilutions in assay buffer were aliquoted in triplicate. To these, 5 *μ*l of diluted enzyme was added, followed by 10 *μ*l of the quenched peptide substrate. The plates were incubated at 37°C until at least 10% substrate turnover or 5 *μ*M product had formed. Fluorescence was measured using a Tecan Safire II (Excitation 320 nm/Emission 420 nm). Each plate included a standard curve of reaction product and positive (no enzyme) and negative (dimethyl sulfoxide vehicle) controls. Dose-response data were plotted and inhibition values (IC_50_) determined by linear regression analysis using GraphPad Prism (GraphPad Software, La Jolla, CA).

#### Animals.

Experiments were performed with 6-to-7-week-old male Wistar rats obtained from Charles River Laboratories (Wilmington, MA). All animals were housed in Saint Louis University facilities under controlled conditions of temperature, humidity, and a 12-hour light/dark cycle. They were maintained on standard rodent chow with free access to water. Animal care and all procedures were performed in accordance with the National Institutes of Health Guide for the Care and Use of Laboratory Animals and approved by the university’s institutional animal care and use committee.

#### Castor Oil–Induced Diarrhea Model.

Rats were randomly assigned to test groups and fasted for 24 hours before the experiment, with free access to water. Animals were weighed and individually housed in metabolic cages containing white paper underneath to collect feces. Test compounds or vehicles were administered by oral gavage (5 ml/kg), and then at indicated times, 10 ml/kg body weight of 100% castor oil was administered by oral gavage to induce diarrhea. Animals had access to food within 5 minutes. Stool weight was recorded starting 15 minutes after castor oil administration and repeated every 15 minutes up to 4 hours. Incidence of diarrhea was recorded as the number of animals in each group exhibiting unformed watery stool. After 4 hours, animals were euthanized with CO_2_, and blood and tissues were collected for analyses.

#### Charcoal Meal Model of Intestinal Motility.

Rats were fasted for 18 hours before the experiment, with free access to water. Animals were weighed, and vehicle or compounds were administered by oral gavage. Since the vehicle used in the castor oil studies was found to have a small but reproducible effect in this assay, 5% ethanol was used since it produced no such effect. After 30 minutes, each animal received standard charcoal meal (10% suspension of activated charcoal in 5% gum arabic) by oral gavage (5 ml/kg). Animals were given access to food within 1 minute and sacrificed 20 minutes after the charcoal meal. The intestines were carefully removed. Motility was calculated as the measured distance traveled by the charcoal as a percentage of total length from pyloric sphincter to ileocecal junction.

#### Compound Measurement in Blood and Tissues.

Plasma and tissue homogenates (prepared as described to follow) were diluted with control naïve rat plasma as appropriate to bring samples into the standard curve range (1–1000 ng/ml). Internal standard was added to all samples and standards before extraction at a 100 ng/ml final concentration. Samples were capped and mixed on a multiplate vortexer for 1 minute. After mixing, 300 *μ*l of acetonitrile was added, vortexed for 10 minutes, and centrifuged for 5 minutes at 3200 rpm. The supernatant was transferred to a 96-well sample plate and capped for liquid chromatography-tandem mass spectrometry analysis using a system consisting of an LC-20AD pump (Shimadzu, Kyoto, Japan), an HTC PAL autosampler (Leap Technologies, Carrboro, NC), and a Sciex API-4000 mass spectrometer in ESI mode (AB Sciex, Foster City, CA). The MRM transitions for thiorphan, LBQ657, candoxatrilat, and UK-505,779 were *m/z*: 252 > 217, 382 > 282, 398 > 278, and 387 > 137, respectively. An Amour C18 reverse-phase column (2.1 × 30 mm, 5 *μ*m; Analytical Sales and Services, Pompton Plains, NJ) was used for chromatographic separation. Mobile phases were 0.1% formic acid (aqueous) and 100% acetonitrile (organic) with a flow rate of 0.35 ml/min. The starting phase was 10% acetonitrile for 0.9 minutes, increased to 90% acetonitrile over 0.4 minutes, maintained an additional 0.2 minutes, returned to 10% acetonitrile over 0.4 minutes, and then held for 1.6 minutes. Peak areas were integrated using Analyst 1.5.1 (AB Sciex, Foster City, CA).

#### NEP Enzyme Activity in Tissues.

Four or five 2-mm Zirconia beads (Biospec, Bartlesville, OK) were added to fresh weighed tissue specimens (50–250 mg). Assay buffer consisting of 50 mM Tris-HCl (pH 7.4), 200 mM NaCl, and 0.05% TritonX-100 was added (4 *μ*l/mg), and the sample was homogenized for 1 minute using a bead beater (Biospec). Stored frozen homogenates were thawed at room temperature and centrifuged 10 minutes at 18,000*g*, and the protein concentration was determined for each supernatant. An appropriate dilution was selected for each tissue, and 10-*μ*l aliquots were dispensed in triplicate to 96-well black half a rea plates (Corning no. 3686). NEP substrate 2Abz-dArg-Gly-Leu-C2-Dnp (American Peptide, Sunnyvale, CA) was prepared at 1 mM in 20% acetonitrile/water and diluted to 100 *μ*M in assay buffer, and 10 *μ*l was dispensed to homogenate-containing wells. The plate was immediately placed in a spectrophotometer in kinetic mode with five reads at 3-minute intervals (Ex320 nm/Em 420 nm). Kinetic data were plotted to determine the rate of product formation by linear regression with interpolation against a standard curve, and the rate was normalized to protein concentration.

#### Statistical Analysis.

Unless specifically noted, statistical analyses were performed using one-way analysis of variance (ANOVA). Least significant difference (LSD) was used for the comparison of means, and *P* values were calculated with GraphPad Prism.

## Results

### 

#### Potency and Selectivity of NEP Inhibitors.

Racecadotril and seven other NEP inhibitors that had previously advanced to human clinical trials for varied disease indications were obtained or synthesized for evaluation in our studies (Table 1; Supplemental Fig. 1). The active metabolite forms of three prodrugs (racecadotril, candoxatril, and sacubitril) were also obtained or synthesized. The available active forms of the compounds were tested for inhibition of recombinant purified human NEP activity in vitro, and each was found to be extremely potent (Table 1). Although NEP has been implicated as a predominant peptidase involved in enkephalin degradation, other peptidases such as aminopeptidase N and puromycin-sensitive aminopeptidase are also reported to have enkephalinase activity (Khaket et al., 2012). Furthermore, altering the activities of angiotensin-converting enzymes (ACEs) and endothelin-converting enzymes or insulin-degrading enzyme would have potential to affect blood pressure or insulin levels, which would not be desirable in diarrheal disease states. Therefore, we evaluated the compounds in a panel of biochemical assays for these peptidases. Three of the NEP inhibitors were also active against ACE; this was expected since these were originally designed to inhibit both enzymes for the treatment of cardiovascular indications (Arnal et al., 2001; Hubner et al., 2001; Rossi, 2003; Davidson et al., 2012). The compounds showing the best combination of high NEP potency (IC_50_ < 0.1 *μ*M) and selectivity (>60-fold) were the active metabolite forms of the prodrugs racecadotril (thiorphan), candoxatril (candoxatrilat), and sacubitril (LBQ657), as well as the non-prodrug UK-505,749.

**TABLE 1:** Peptidase potency and selectivity of neutral endopeptide (NEP) inhibitors used in this study

Compound	Indication	Clinical Phase	NEP*^a^*	ACE*^a^*	ECE-1*^a^*	ECE-2*^a^*	IDE*^a^*	APN*^a^*	PSA*^a^*
Racecadotril*^b^*	Diarrhea	Launched	0.02	10.2	>100	>100	>100	>100	>100
Candoxatril*^b^*	Cardiovascular	Phase 3 (halted)	0.06	>100	3.9	10	>100	29	>100
Sacubitril*^b^*	Cardiovascular	Launched (LCZ696)	0.02	>100	38	47	>100	14	>100
UK-505,749	Genitourinary	Phase 1 (halted)	0.004	>100	3.3	15	>100	>100	>100
Omapatrilat	Cardiovascular	Phase 3 (halted)	0.04	0.19	21	86	>100	28	>100
Gemopatrilat	Cardiovascular	Phase 1 (halted)	0.06	0.23	12	77	>100	18	18
Ilepatril*^c^*	Cardiovascular	Phase 2 (halted)	NA	NA	NA	NA	NA	NA	NA
MDL 100240*^c^*	Cardiovascular	Phase 2 (halted)	NA	NA	NA	NA	NA	NA	NA

ACE, angiotensin-converting enzyme; APN, aminopeptidase N; ECE, endothelin-converting enzyme; IDE, insulin-degrading enzyme; PSA, puromycin-sensitive aminopeptidase.

^a^ Mean IC_50_ value (*μ*M).

^b^ Peptidase activity is for the active form of this prodrug.

^c^ The active form of this prodrug was not available (NA) for testing.

#### Efficacy in Castor Oil–Induced Experimental Diarrhea.

**Fig. 1.**
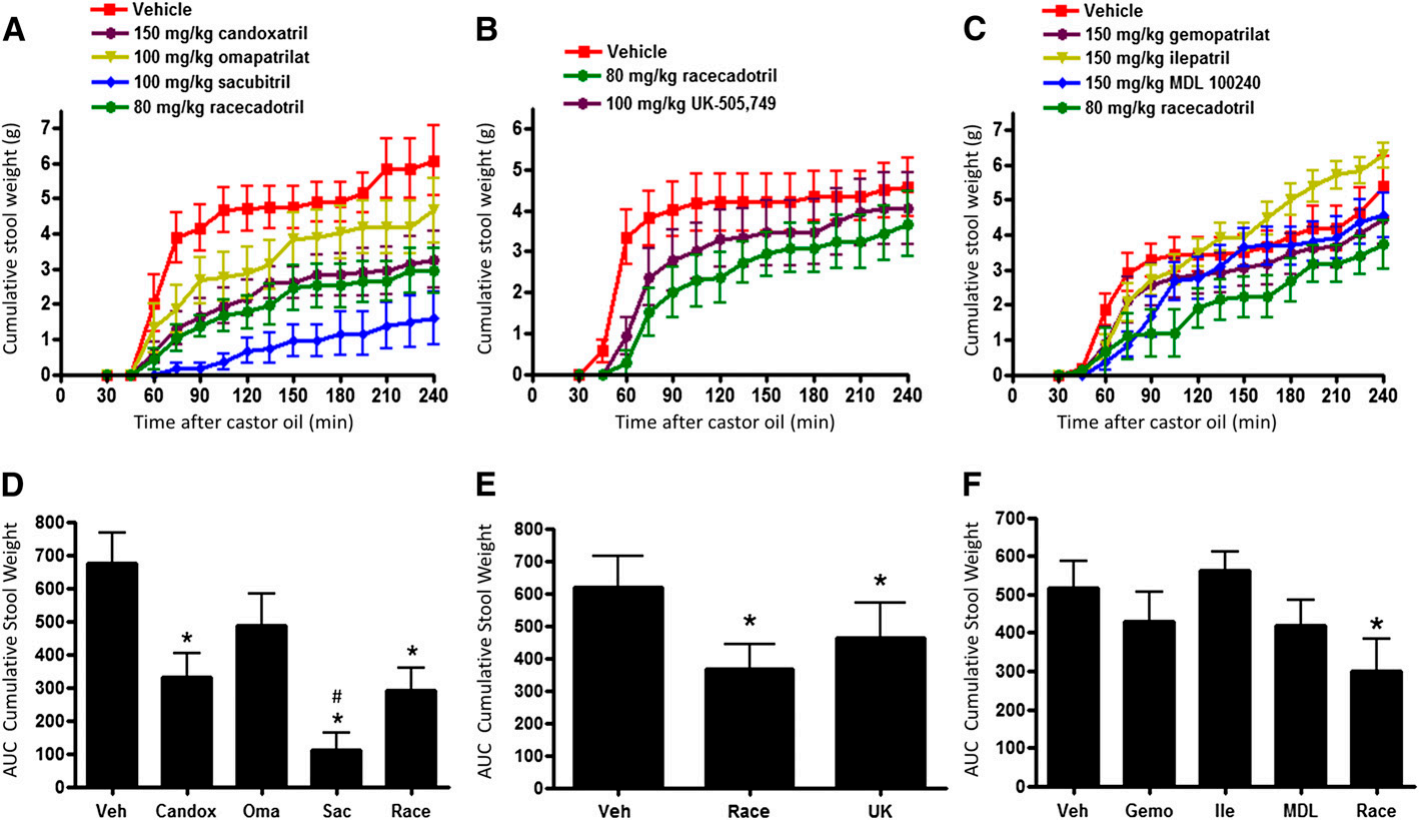
Effect of NEP inhibitor treatments on castor oil–induced experimental diarrhea. Rats were given a single oral treatment of vehicle (10:10:80 ethanol/kolliphor EL/water) or NEP inhibitor 30 minutes before castor oil administration as described in *Materials and Methods*. (A–C) Time course of cumulative stool weight measured in independent studies of racecadotril and other indicated NEP inhibitors. (D and F) Total area under the time course curves (AUC) calculated for each treatment from the stool accumulation data. Data are expressed as the mean ± S.E.M. (*n* = 10/group; owing to gavage injury, one animal was terminated early from both the vehicle and racecadotril groups shown in (A); these were excluded from the analysis). *P < 0.05 versus vehicle. ^#^*P* < 0.05 versus racecadotril (two-tailed T test).

Because racecadotril was previously shown to be efficacious in both rat and human studies of castor oil–induced diarrhea (Marcais-Collado et al., 1987; Baumer et al., 1992), we performed studies to determine whether our other assembled NEP inhibitors would also have efficacy in this model. In each study, racecadotril was employed as a positive control and was administered orally in a single dose selected for maximal efficacy based on a previous report (Marcais-Collado et al., 1987) and our own dose-ranging studies (data not shown). The dose level for other agents was selected to fall within the safe and effective range previously used in other disease models. When a single dose of agent was orally administered to fasted rats 30 minutes before castor oil delivery, the highly potent and selective NEP inhibitors racecadotril, candoxatril, sacubitril, and UK-505,749 each significantly reduced cumulative stool output over the next 4 hours (*P* < 0.05) (Fig. 1). Treatment with sacubitril appeared particularly effective and was the only agent significantly superior to racecadotril (*P* ≤ 0.05). Furthermore, sacubitril was the only agent that also reduced the incidence of diarrhea; only 40% (4/10) of treated animals produced any loose stool during the 4-hour observation period, whereas at least one loose stool was seen in 90% to 100% of rats in all other treatment groups.

#### Comparison of Dose-Response Relationships.

**Fig. 2.**
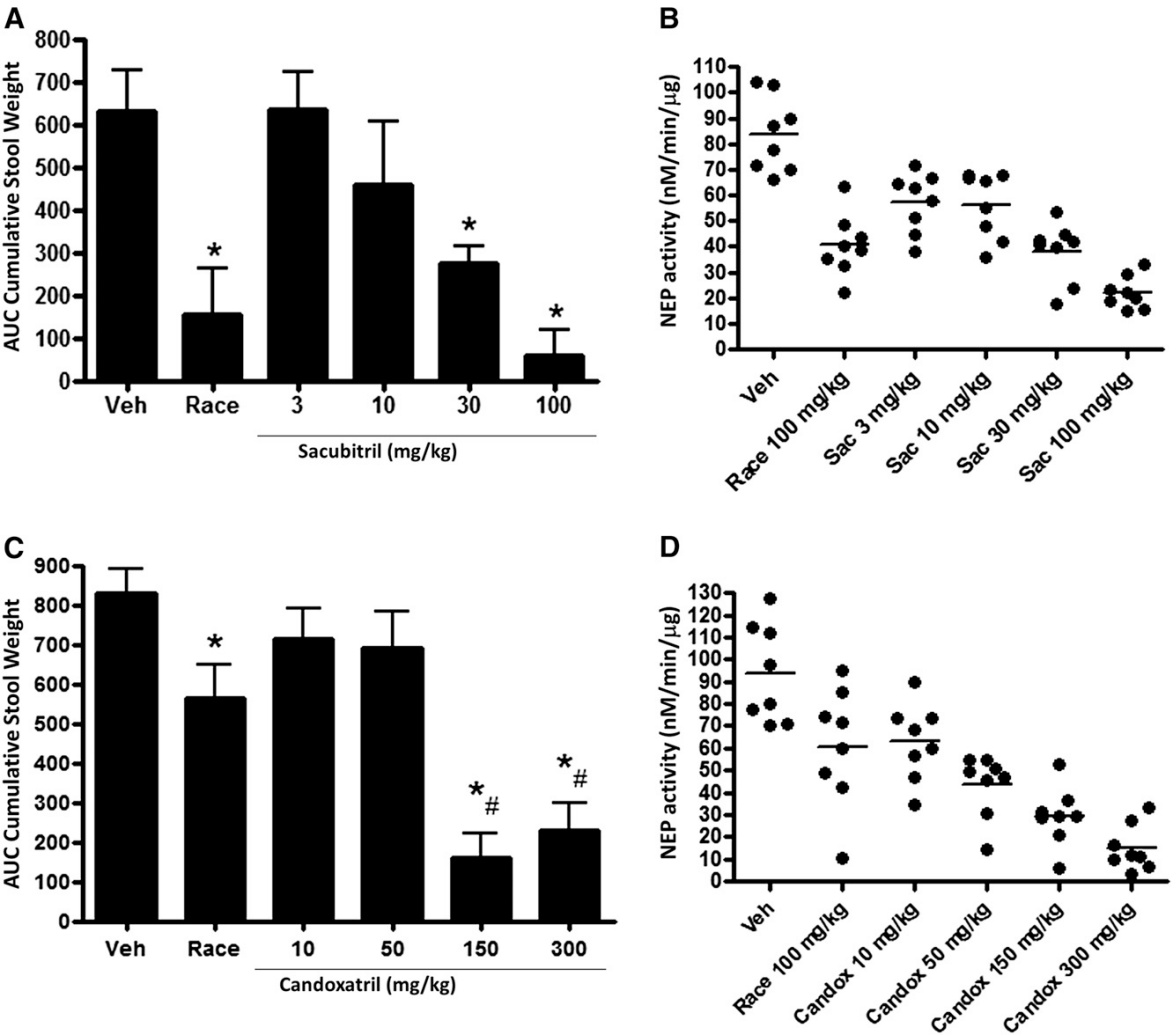
Dose-dependent efficacy of select NEP inhibitors in experimental diarrhea. (A) Diarrhea measured in rats given a single oral treatment with vehicle (10:10:80 ethanol/kolliphor EL/water), 100 mg/kg racecadotril, or varying doses of sacubitril 30 minutes before castor oil administration. Data are expressed as the mean ± S.E.M (*n* = 8/group). *P < 0.05 versus vehicle. (B) Dose effect of sacubitril treatment on NEP enzyme activity measured in lung tissue extracts, with racecadotril comparator. Values are shown for each animal in the treatment group; mean is indicated by the horizontal line. (C) Diarrhea measured in rats given a single oral treatment with 100 mg/kg racecadotril or varying doses of candoxatril 30 minutes before castor oil administration. Data are expressed as the mean ± S.E.M. (*n* = 8/group). *P < 0.05 versus vehicle, ^#^*P* < 0.05 versus racecadotril. (D) Dose effect of candoxatril treatment on NEP enzyme activity measured in lung tissue extracts made from each animal, with racecadotril comparator.

The effects of sacubitril and candoxatril in castor oil–treated rats were examined in more detail since both these agents had previously advanced to phase 3 clinical development for other indications. Sacubitril reduced stool output in a dose-dependent manner (Fig. 2A), and diarrhea was almost completely prevented at the highest tested dose (25% incidence). Racecadotril also significantly reduced stool output but did not prevent the occurrence of diarrhea (100% incidence). We also measured NEP enzyme activity in lung tissue samples from these animals at the end of the study (Fig. 2B). Our pilot studies had found that protein-normalized basal NEP activity was easily detected in the lung, kidney, and small intestine; activity in plasma was undetectable and was extremely low in the brain and colon. Because administration of castor oil makes subsequent processing of intestinal tissue difficult, we analyzed lung tissue as a surrogate tissue to assess pharmacologic inhibition of the drug target. The effect of sacubitril treatment on NEP activity in lung tissue corresponded well with the measurement of stool output. We conducted a similar study with candoxatril and found a strong, nearly equal reduction of stool output at the two highest doses (Fig. 2C). Racecadotril again was efficacious in this study, although not nearly so much as seen in the study with sacubitril. Dosage of candoxatril was inversely correlated with NEP enzyme activity in lung tissues (Fig. 2D). Mean suppression of NEP activity by racecadotril was less and more variable in the candoxatril dose-response study than in the sacubitril dose-response study, which likely accounts for the apparent difference in racecadotril efficacy between these studies.

**Fig. 3.**
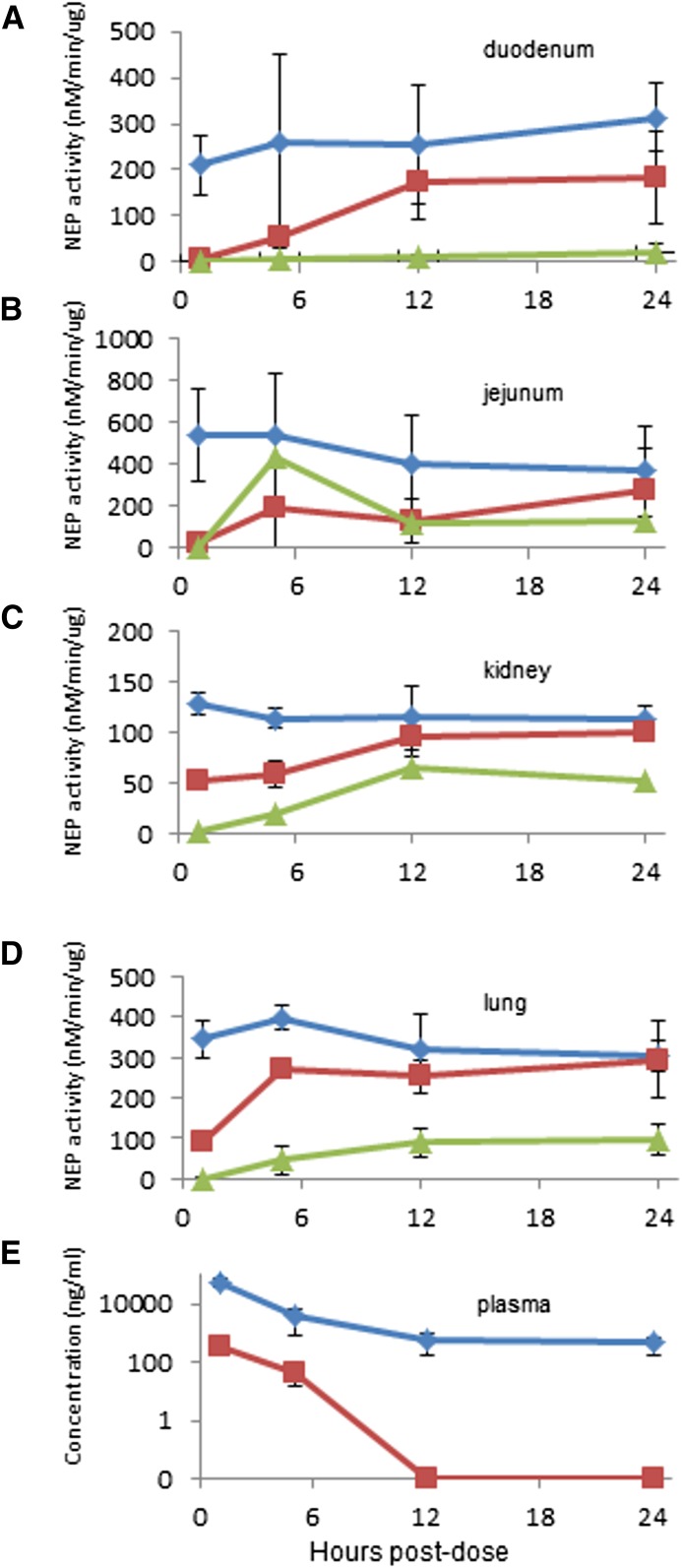
PK and PD comparison of sacubitril and racecadotril. Vehicle (10:10:80 ethanol/kolliphor EL/water) (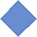), 100 mg/kg racecadotril (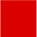), or 100 mg/kg sacubitril (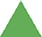) was administered to normal healthy rats by oral gavage. At each time point, samples were collected from each indicated organ tissue for measurement of NEP enzyme activity. (E) At the same time points, the active metabolites of racecadotril (thiorphan, 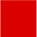) and sacubitril (LBQ657, 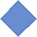) were determined in plasma samples as described in *Materials and Methods*. Data are expressed as the mean ± S.E.M of values from four rats.

To better assess the PK and PD characteristics of sacubitril in multiple tissues, including segments of the small intestine, plasma and tissues samples were collected from normal healthy (i.e., not treated with castor oil) rats at 1, 5, 12, and 24 hours after a single oral 100-mg/kg dose of this drug or a 100-mg/kg dose of racecadotril as a comparator. The results show both sacubitril and racecadotril suppress NEP enzyme activity to barely detectable levels in the duodenum and jejunum 1 hour after dosing, but sacubitril sustains this inhibition for a much longer period than racecadotril, even up to 24 hours (Fig. 3, A and B). In lung and kidney tissue, sacubitril produced a much greater magnitude of NEP inhibition than racecadotril after 1 hour, and its effect was still apparent after 24 hours, whereas NEP activity had returned to normal in racecadotril-treated animals (Fig. 3, C and D). Determination of the average drug concentration in plasma revealed that the active metabolite of sacubitril (LBQ657) was present at much higher levels than that found for the active metabolite of racecadotril (thiorphan) (Fig. 3E). Tissue exposures for the active forms were also 10–100 times higher for sacubitril than racecadotril (Supplemental Fig. 2). Therefore, drug concentration and the effect on the target appear to be correlated, with sacubitril producing a greater magnitude and duration of NEP inhibition versus racecadotril over a 24-hour period when both are orally administered at an equivalent dose.

In a separate study using fasted rats, we compared the PD effect of all four NEP inhibitors with antidiarrheal efficacy at two time points: 1 and 5 hours post-treatment. These data are shown in Supplemental Fig. 3 and are summarized in Table 2. Every compound showed a very strong suppression of enzyme activity in the intestinal tissues at 1 hour; however, sacubitril, candoxatril, and UK-505,749 produced more marked systemic NEP inhibition (i.e., kidney and lung) than racecadotril at this time point. At 5 hours postdose, these three compounds showed continued strong inhibition of NEP in the intestinal segments (>92%), whereas activity had partially recovered in racecadotril-treated animals. Measurement of plasma concentrations of the active form of each compound showed that at 5 hours, levels of thiorphan (racecadotril) and candoxatrilat (candoxatril) were roughly similar, whereas those of LBQ657 (sacubitril) and UK-505,749 were 5- to 10-fold higher (Supplemental Table 1). Thiorphan measured in duodenum, lung, and kidney tissues appeared substantially lower than other tested compounds at this time point.

**TABLE 2:** Relative neutral endopeptidase (NEP) inhibition in tissues after single dose oral treatments of normal rats

	Race 1 h	Candox 1 h	Sac 1 h	UK 1 h	Race 5 h	Candox 5 h	Sac 5 h	UK 5 h
Kidney	56%	99%	100%	99%	39%	45%	90%	86%
Lung	75%	98%	99%	99%	59%	52%	79%	94%
Duodenum	98%	100%	100%	100%	85%	97%	99%	99%
Jejunum	90%	100%	99%	100%	32%	92%	97%	98%

NEP inhibitors were administered to normal healthy rats by oral gavage at the following doses: racecadotril (Race) 100 mg/kg, candoxatril (Candox) 150 mg/kg, sacubitril (Sac) 100 mg/kg, and UK-505,749 (UK) 100 mg/kg. Tissue lysates were prepared after 1 and 5 h, and NEP activity was measured as described in *Materials and Methods*. Percentage inhibition is the relative reduction in activity compared with vehicle-treated control animals.

**Fig. 4.**
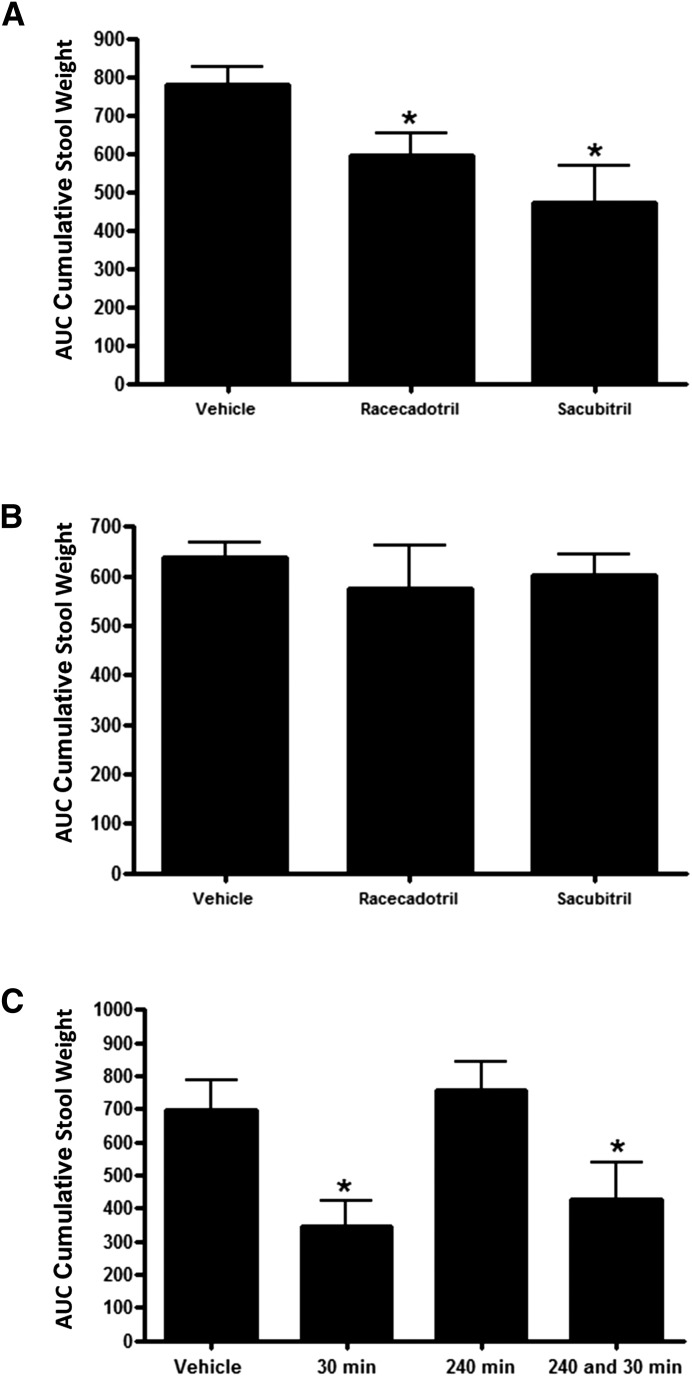
Effect of compound predosing interval on protection from castor oil–mediated diarrhea. (A) Diarrhea measured in rats given a single oral treatment with 100 mg/kg racecadotril or 100 mg/kg sacubitril 90 minutes before castor oil administration. (B) Diarrhea measured in rats given a single oral treatment with 100 mg/kg racecadotril or 100 mg/kg sacubitril 150 minutes before castor oil administration. (C) Diarrhea measured in rats treated with sacubitril at 30 minutes, 240 minutes, or both 30 and 240 minutes before castor oil administration. Data are expressed as the mean ± S.E.M. [*n* = 8/group; one animal was terminated early from the vehicle group in (C) owing to gavage injury, and it was excluded from the analysis]. *P < 0.05 versus vehicle.

Our previous studies had all been conducted with dosing of test compounds 30 minutes before administration of the castor oil stimulus. We reasoned that the long duration of NEP inhibition mediated by sacubitril may allow expansion of this interval. Sacubitril treatment 90 minutes before castor oil administration did reduce stool output further than did racecadotril treatment (Fig. 4A), but the difference in effect between the two drugs was not statistically significant. Neither drug was effective when administered 150 or 240 minutes before castor oil (Fig. 4, B and C). When a group of rats was pretreated with sacubitril at 240 minutes, and then the same group was treated again 30 minutes before castor oil, efficacy was similar to rats that had received only the 30-minute pretreatment (Fig. 4C). Furthermore, consistent with the time-course analysis described here in animals not treated with castor oil, NEP activity in tissues was similarly reduced in all groups at the end of the study, regardless of when first treatment was initiated relative to castor oil administration (Supplemental Fig. 4). These results suggest that the lack of antidiarrheal effect observed with the longer pretreatment intervals is not due to internalization or desensitization of opioid receptors.

#### Intestinal Motility.

**Fig. 5.**
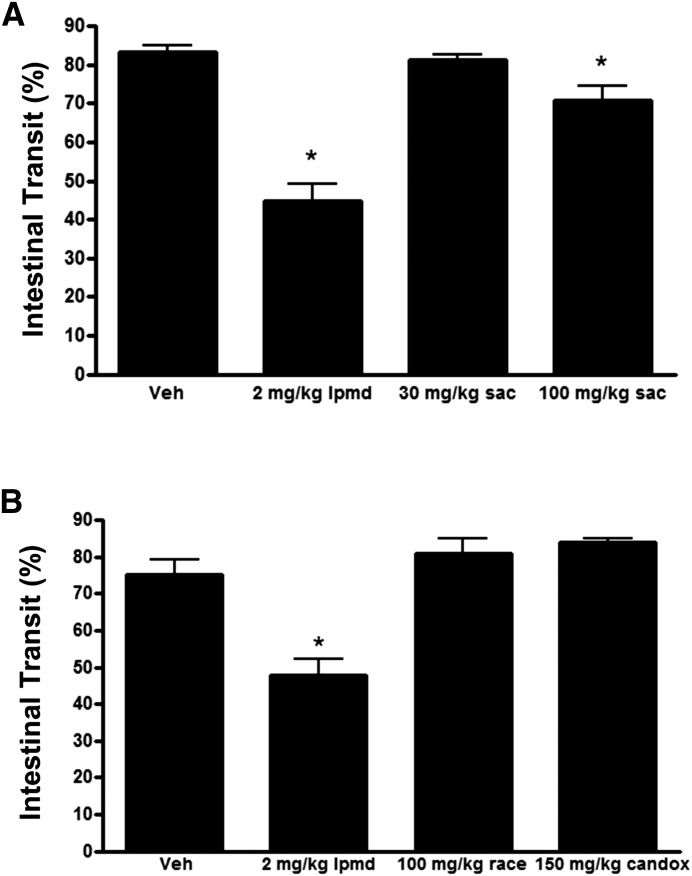
NEP inhibitor treatments do not delay intestinal transit. (A) Effects of loperamide and sacubitril treatments on gastrointestinal transit as evaluated using the charcoal meal test in rats. (B) Effects of loperamide, racecadotril, and candoxatril treatments on gastrointestinal transit in the same model. Animals received test agents 30 minutes before administration of the charcoal meal and were sacrificed 20 minutes later. Vehicle was 5% ethanol in water. The racecadotril formulation also contained 0.2% Tween 20 for solubility. Data are expressed as the mean ± S.E.M. of values from five rats. *P < 0.05 versus vehicle.

The antidiarrheal effects of racecadotril are thought to be mediated predominantly through an antisecretory mode of action. This is supported by experimental data showing this drug, unlike μ agonists such as loperamide, did not delay gastrointestinal motility in the commonly used charcoal meal test (Marcais-Collado et al., 1987). To determine the effect of sacubitril in this model, we treated fasted rats 30 minutes before administration of a charcoal meal and then measured the effect on the distance traveled in 20 minutes. The higher dose of sacubitril produced a significant but very modest effect at 100 mg/kg dose and no effect at 30 mg/kg (Fig. 5A). In contrast, loperamide greatly slowed transit compared with both the vehicle and sacubitril treatment groups. A separate study showed racecadotril and candoxatril had no effect on intestinal motility in this model (Fig. 5B).

## Discussion

Multiple small, randomized single- or double-blinded, placebo-controlled studies have been conducted with racecadotril in child and adult patient populations with various forms of acute diarrhea (Matheson and Noble, 2000; Lehert et al., 2011; Eberlin et al., 2012). Most judged the treatment at least modestly successful, and comparisons between loperamide and racecadotril suggested similar efficacy, with racecadotril having less risk of adverse events such as rebound constipation (Prado, 2002; Wang et al., 2005; Eberlin et al., 2012). Demonstration of racecadotril’s clinical benefit for ASD was preceded by abundant preclinical characterization of its efficacy in the rat castor oil model of secretory diarrhea. Importantly, in a small controlled human trial with adult volunteers, racecadotril significantly decreased castor oil–induced diarrhea (Baumer et al., 1992). Based on all existing data with racecadotril, we contend that the rat castor oil model appears highly relevant for the evaluation of other NEP inhibitors and has an excellent likelihood of being predictive of ASD clinical efficacy for this mechanistic class.

The results from our current report show several of the highly specific NEP inhibitors that were advanced to clinical trials for other indications were as effective or more effective than racecadotril in the rat castor oil model. Interestingly, the NEP/ACE mixed inhibitors we tested were less effective than the more selective NEP inhibitors, despite some having excellent biochemical potency on the NEP target in vitro. Besides enkephalins, NEP and ACE are each capable of hydrolysis of a number of peptide hormones—some of which have prosecretory activities such as VIP and substance P (Skidgel and Erdos, 2004; Leuchte et al., 2015). Perhaps mixed inhibition of these enzymes produces a different profile of stabilized peptides that dampens the effect of selective NEP inhibition in this model. Regardless of the mechanism, the data from our model suggest that no class advantage is conferred in diarrhea treatment with ACE/NEP inhibitors.

The NEP inhibitor that showed the strongest and most consistent efficacy in our studies was sacubitril. Indeed, it was the only tested agent that significantly reduced the incidence of diarrhea resulting from castor oil administration in addition to cumulative stool output. Sacubitril, also known as AHU-377, is one of the two drug components of the combination drug LCZ696 (Entresto), which recently received regulatory approval from the United States Food and Drug Administration for the treatment of heart failure. Candoxatril and UK-505,749, two other NEP inhibitors that were previously evaluated for safety and effectiveness in human clinical trials, also demonstrated promising activity in our model, but their activity was less dramatic than that of sacubitril.

Human PK data for sacubitril, candoxatril, UK-505,797, and racecadotril show the active forms of these compounds are eliminated from plasma with a mean half-life of approximately 11 hours, 16 hours, 6 hours, and 3 hours, respectively (McDowell et al., 1997; Matheson and Noble, 2000; Gu et al., 2010). Our PD analyses showed all three next-generation NEP inhibitors quickly suppressed enzyme activity in intestinal and nonintestinal tissues to a greater magnitude than racecadotril and sustained this inhibition for a longer period. At 5 hours postadministration, sacubitril and UK-505,797 showed continued strong NEP inhibition in intestinal segments (≥97%), whereas activity had partially recovered with candoxatril treatment and even more so with racecadotril treatment. Across all the tissues at 5 hours postadministration, measured NEP inhibition was greatest with the sacubitril and UK-505,797 treatments, followed by candoxatril, and lastly by racecadotril. Plasma and tissue concentrations of the active forms were well correlated with the enzyme inhibition at this time point. These data clearly show the superior PK/PD characteristics of these drugs compared with racecadotril; if our findings can be translated to humans, they may enable less frequent oral dosing than racecadotril, which is given three times daily in the standard regimen.

Most of our diarrhea experiments were conducted with 30 minutes NEP inhibitor predosing relative to the castor oil administration, followed by a 4-hour observation period for measurement of stool output. Because our PK/PD studies showed sacubitril provided nearly complete suppression of NEP activity in intestinal tissues for several hours, we expected that rats may be protected from diarrhea when the interval between NEP inhibitor and castor oil administration was extended. Instead, we found that efficacy was diminished with 90-minute predosing and absent with 150-minute or 240-minute predosing. We hypothesized that this could be due to either 1) desensitization of opioid receptors via receptor internalization as has been previously described (Galligan and Akbarali, 2014) or 2) failure to maintain a sufficient minimum compound exposure threshold at a presumed intestinal compartment, which would be necessary to block the action of the acute castor oil stimulus. Our data in Fig. 4C (showing a 240-minute predose did not block efficacy conferred by a second 30-minute pre-dose) suggest that the opioid system remains fully responsive, consistent with the latter hypothesis.

The requirement for severe acute NEP inhibition for efficacy in this model may be due to the severely acute action of the castor oil stimulus. Intestinal lipases act on ingested castor oil to release ricinoleic acid, which then acts through prostanoid receptors to induce the secretomotor reflex, resulting in diarrhea (Tunaru et al., 2012). NEP inhibitor treatment proximal to castor oil may poise accumulated enkephalins to countermand these effects, but if the drug treatment is administered too early, the recovery of even a small amount of NEP activity in the microenvironment of the enkephalinergic inhibitory synapse may diminish efficacy. Our measures of NEP activity cannot discern the kinetics of drug exposure in subcompartments such as the intestinal epithelium, where compounds are absorbed, and the synaptic clefts. Enkephalins are normally degraded within minutes of release by enteric neurons. It is possible that other NEP substrates such as VIP and substance P are also stabilized by NEP inhibitor treatment so that the net balance of these prosecretory molecules relative to enkephalins at the critical site changes quickly with time. We attempted to study this pharmacology in other rodent models such as cholera toxin–induced secretion in intestinal loops and rotavirus-induced diarrhea but did not observe efficacy (manuscript in preparation), even though racecadotril has been reported to have clinical efficacy for diarrhea of varied origin (Matheson and Noble, 2000; Salazar-Lindo et al., 2000; Eberlin et al., 2012).

Like racecadotril, the newer clinical NEP inhibitors had little or no effect on intestinal motility in the charcoal meal test. Therefore, it seems likely that the clinical application of these compounds for diarrhea patients would, like racecadotril, have a lower risk of adverse events associated with antimotility agents such as loperamide. Furthermore, both sacubitril (as a component of LCZ696) and candoxatril have already been tested in large patient populations in phase 3 cardiovascular clinical trials so that their safety profiles are well known, albeit primarily in adults. The efficacy and pharmacology data from our studies suggest these newer clinical-stage NEP inhibitors originally developed as therapies for other indications may offer advantages over racecadotril for ASD therapy, such as less frequent dosing and potentially improved efficacy. They have the potential for rapid advancement to clinical evaluation for this important indication.

## Supplementary Material

Data Supplement

## Abbreviations

ACE: angiotensin-converting enzyme
AHU-377/sacubitril: 4-(((2*S*,4*R*)-1-([1,1′-biphenyl]-4-yl)-5-ethoxy-4-methyl-5-oxopentan-2-yl)amino)-4-oxobutanoic acid
ASD: acute secretory diarrhea
LBQ657: (2*R*,4*S*)-5-([1,1′-biphenyl]-4-yl)-4-(3-carboxypropanamido)-2-methylpentanoic acid
MDL 100240: (4*S*,7*S*,12b*R*)-7-((*S*)-2-(acetylthio)-3-phenylpropanamido)-6-oxo-1,2,3,4,6,7,8,12b-octahydrobenzo[*c*]pyrido[1,2-*a*]azepine-4-carboxylic acid
NEP: neutral endopeptidase
PK: pharmacokinetic
PD: pharmacodynamic
UK-505,749: (*R*)-2-methyl-3-(1-((3-(2-methylbenzo[*d*]thiazol-6-yl)propyl)carbamoyl)cyclopentyl)propanoic acid
VIP: vasoactive intestinal peptide
