# Supplementary material for: Pharmacologic Comparison of Clinical Neutral Endopeptidase Inhibitors in a Rat Model of Acute Secretory Diarrhea
Source: J Pharmacol Exp Ther. 2016 May;357(2):423–31. doi: 10.1124/jpet.115.231167 (PMC4851326; doi:10.1124/jpet.115.231167)
Supplement: Data Supplement [file supp_357_2_423__index.html]

Pharmacologic Comparison of Clinical Neutral Endopeptidase Inhibitors in a Rat Model of Acute Secretory Diarrhea — Pharmacologic Comparison of Clinical Neutral Endopeptidase Inhibitors in a Rat Model of Acute Secretory Diarrhea — Comparative Effects of NEP Inhibitors on Diarrhea in Rats — Data Supplement 

# Pharmacologic Comparison of Clinical Neutral Endopeptidase Inhibitors in a Rat Model of Acute Secretory Diarrhea

## Data Supplement

**Files in this Data Supplement:**

- Supplemental Data - 4 supplemental figures, 1 table.
