# Supplementary material for: Pharmacologic Comparison of Clinical Neutral Endopeptidase Inhibitors in a Rat Model of Acute Secretory Diarrhea
Source: J Pharmacol Exp Ther. 2016 May;357(2):423–31. doi: 10.1124/jpet.115.231167 (PMC4851326; doi:10.1124/jpet.115.231167)
Supplement: Data Supplement [file supp_115.231167_231167_suppl_data.pdf]

Pharmacologic Comparison of Clinical Neutral Endopeptidase Inhibitors in a Rat Model of Acute Secretory Diarrhea

David W. Griggs, Michael J. Prinsen, Jonathan Oliva, Mary A. Campbell, Stacy D. Arnett, Deena Tajfirouz, Peter G. Ruminski, Ying Yu, Brian Bond, Yuhua Ji, Georg Neckermann, Robert K.M. Choy, Eugenio de Hostos, and Marvin J. Meyers

Journal of Pharmacology and Experimental Therapeutics

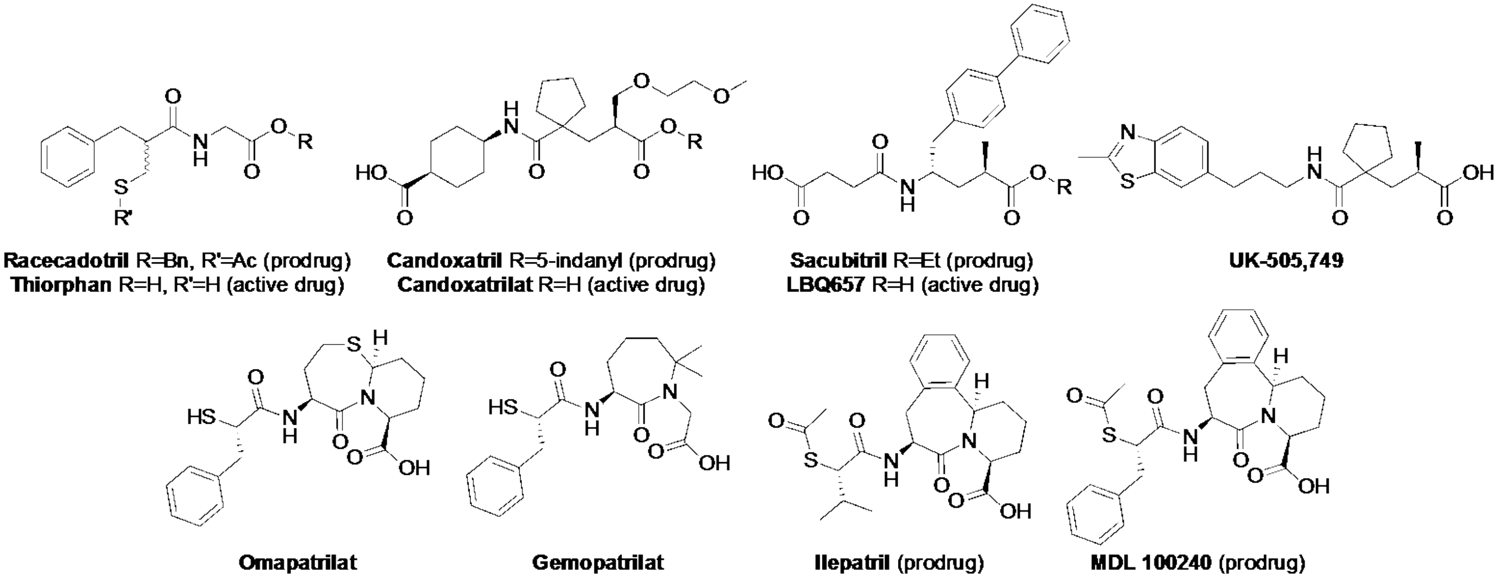

Supplementary Figure 1. Chemical structures of compounds used in this study.

Pharmacologic Comparison of Clinical Neutral Endopeptidase Inhibitors in a Rat Model of Acute Secretory Diarrhea

David W. Griggs, Michael J. Prinsen, Jonathan Oliva, Mary A. Campbell, Stacy D. Arnett, Deena Tajfirouz, Peter G. Ruminski, Ying Yu, Brian Bond, Yuhua Ji, Georg Neckermann, Robert K.M. Choy, Eugenio de Hostos, and Marvin J. Meyers

Journal of Pharmacology and Experimental Therapeutics

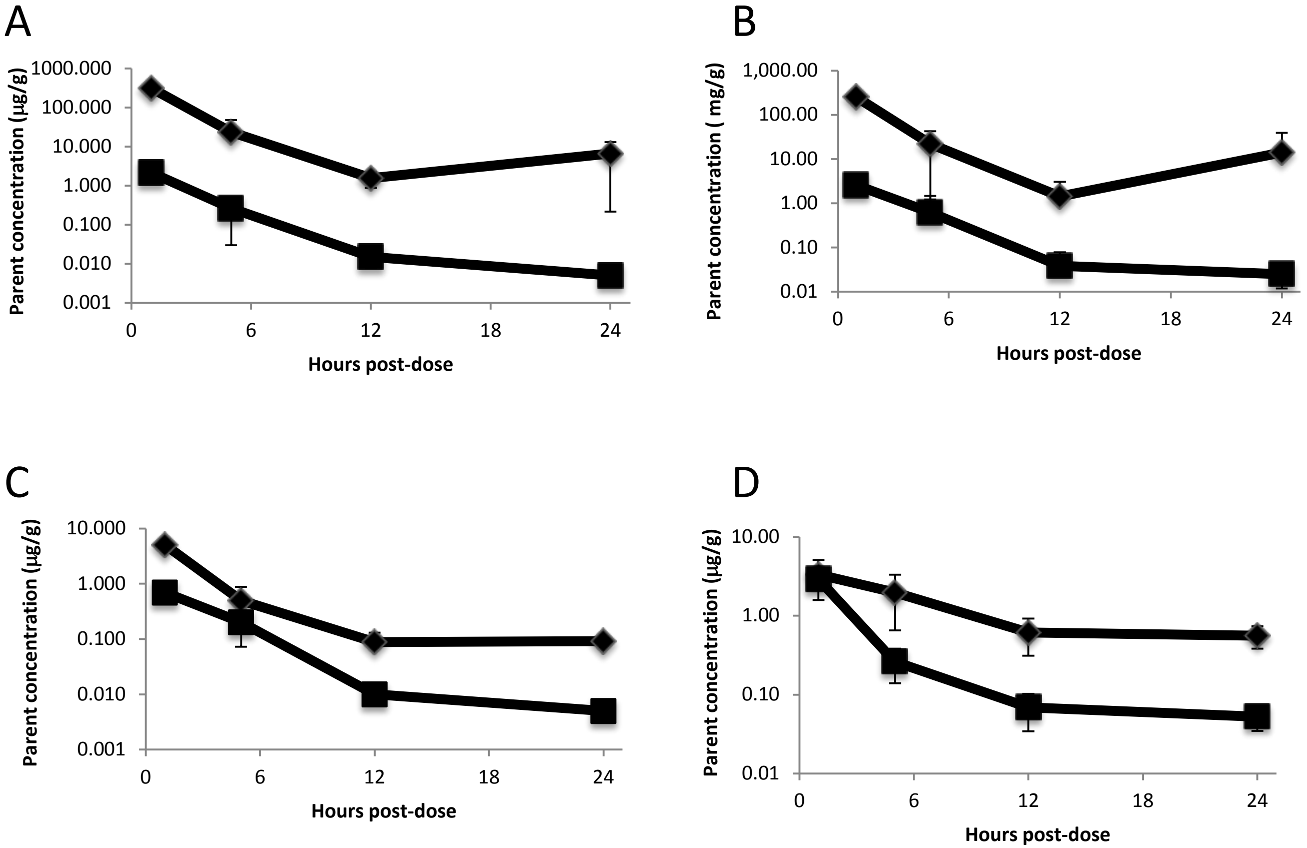

Supplementary Figure 2. Tissue pharmacokinetic profiles of sacubitril and racecadotril. 100 mg/kg racecadotril or 100 mg/kg sacubitril was administered to normal healthy rats by oral gavage. At the indicated time points, tissue samples were collected and the concentration of each drug's active metabolite was measured by mass spectroscopy. (t) LBQ657 (parent of sacubitril); (n) thiorphan (parent of racecadotril). Data are expressed as the mean ± S.E.M. of values from four mice. (A) duodenum, (B) jejunum, (C) lung, *(D)* kidney.

Pharmacologic Comparison of Clinical Neutral Endopeptidase Inhibitors in a Rat Model of Acute Secretory Diarrhea

David W. Griggs, Michael J. Prinsen, Jonathan Oliva, Mary A. Campbell, Stacy D. Arnett, Deena Tajfirouz, Peter G. Ruminski, Ying Yu, Brian Bond, Yuhua Ji, Georg Neckermann, Robert K.M. Choy, Eugenio de Hostos, and Marvin J. Meyers

Journal of Pharmacology and Experimental Therapeutics

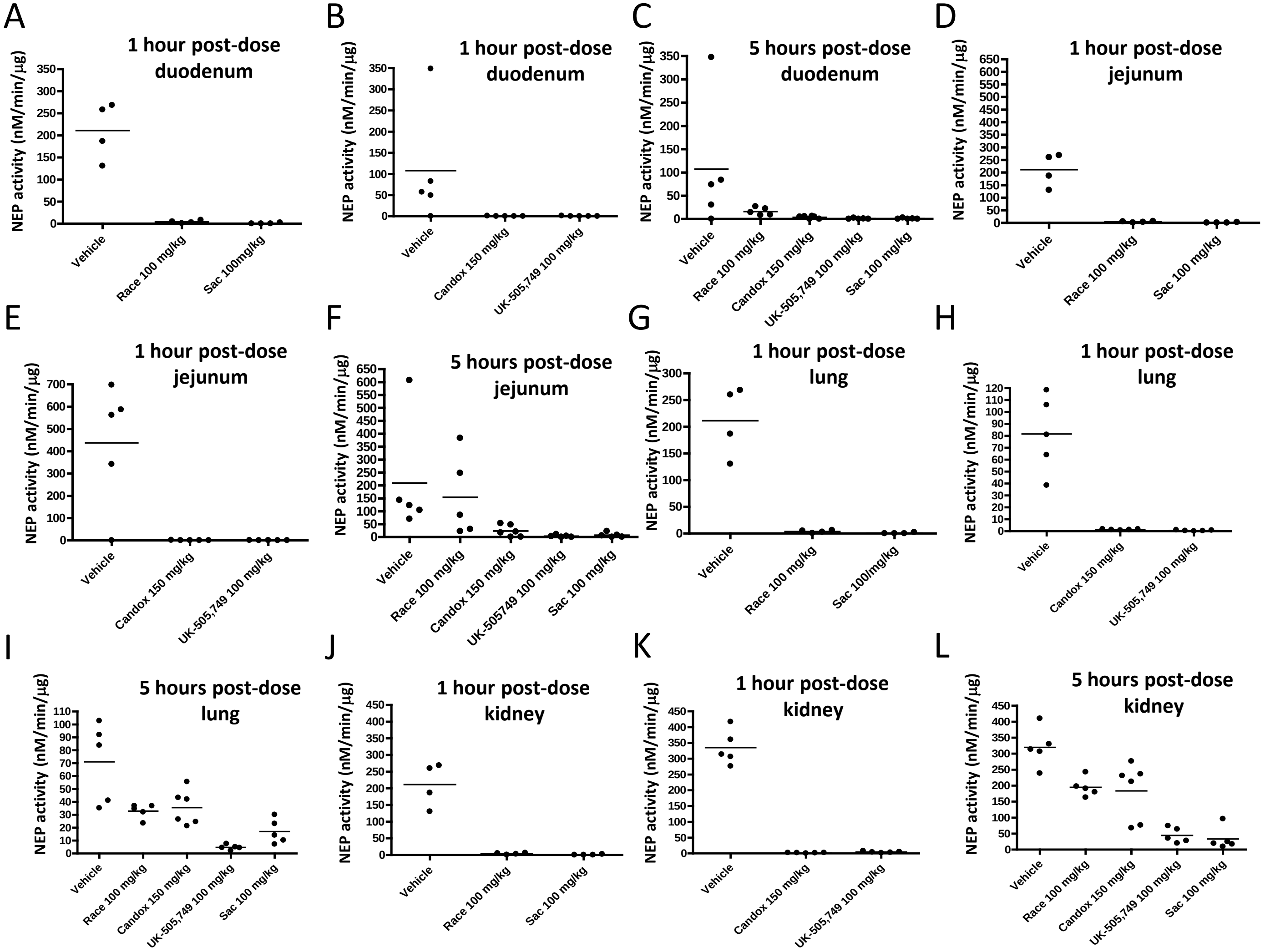

Supplementary Figure 3. NEP inhibition in tissues after single-dose oral treatments of normal rats. Vehicle (10/10/80 ethanol/kolliphoreEL/water) or NEP inhibitors were administered to normal healthy rats by oral gavage at the indicated dose. At 1 and 5 hours post-dose, samples were collected for measurement of NEP enzyme activity in (A–C) duodenum, (D–F) jejunum, (G–I) lung, and (J–L) kidney. Values are shown for each animal in the treatment group, with the mean indicated by the horizontal line. Data from A, D, G, and J were obtained from a different study than B, C, E, F, H, J, K, and L.

Pharmacologic Comparison of Clinical Neutral Endopeptidase Inhibitors in a Rat Model of Acute Secretory Diarrhea

David W. Griggs, Michael J. Prinsen, Jonathan Oliva, Mary A. Campbell, Stacy D. Arnett, Deena Tajfirouz, Peter G. Ruminski, Ying Yu, Brian Bond, Yuhua Ji, Georg Neckermann, Robert K.M. Choy, Eugenio de Hostos, and Marvin J. Meyers

Journal of Pharmacology and Experimental Therapeutics

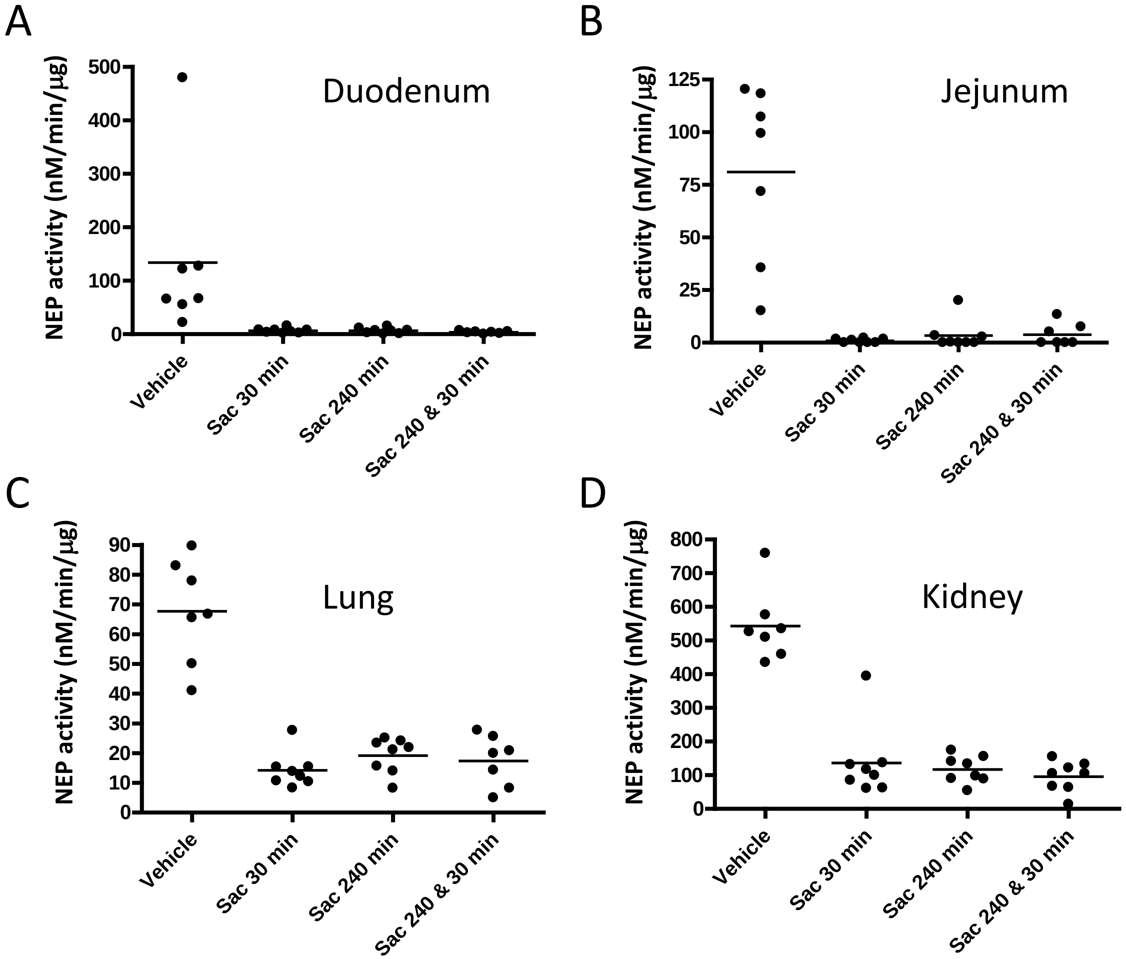

Supplementary Figure 4. Effect of variable compound pre-dosing periods on terminal NEP activity in the castor oil diarrhea model. NEP enzyme activity was measured in tissues dissected from rats treated with sacubitril at 30 minutes, at 240 minutes, or at both 30 and 240 minutes prior to castor oil administration. The control group received vehicle (10/10/80 ethanol/kolliphoreEL/water) at both 30 and 240 minutes prior to castor oil. Values are shown for each animal in the treatment group, with the mean indicated by the horizontal line.

Pharmacologic Comparison of Clinical Neutral Endopeptidase Inhibitors in a Rat Model of Acute Secretory Diarrhea

David W. Griggs, Michael J. Prinsen, Jonathan Oliva, Mary A. Campbell, Stacy D. Arnett, Deena Tajfirouz, Peter G. Ruminski, Ying Yu, Brian Bond, Yuhua Ji, Georg Neckermann, Robert K.M. Choy, Eugenio de Hostos, and Marvin J. Meyers

Journal of Pharmacology and Experimental Therapeutics

Supplementary Table 1
Mean concentrations of NEP inhibitor parent compounds 5 hours post-dose[a]

	**Thiorphan**	**LBQ657**	**Candoxatrilat**	**UK-505,749**
Plasma (ng/ml)	138	1157	82	855
Duodenum (μg/g)	0.6	3.2	7.9	2.0
Jejunum (μg/g)	0.4	0.4	0.4	2.0
Lung (μg/g)	0.8	6.0	55	13
Kidney (μg/g)	0.3	4.6	11	14

[a]NEP inhibitors were administered to rats by oral gavage at the following doses: racecadotril 100 mg/kg, sacubitril 100 mg/kg, candoxatril 150 mg/kg, and UK-505,749 100 mg/kg. The active parent forms of the compounds were measured 5 hours later by mass spectroscopy as described in Materials and Methods.
